# Polymer Electrolyte Membranes Containing Functionalized Organic/Inorganic Composite for Polymer Electrolyte Membrane Fuel Cell Applications

**DOI:** 10.3390/ijms232214252

**Published:** 2022-11-17

**Authors:** Seansoo Hwang, HyeonGyeong Lee, Yu-Gyeong Jeong, Chanhee Choi, Inhyeok Hwang, SeungHyeon Song, Sang Yong Nam, Jin Hong Lee, Kihyun Kim

**Affiliations:** 1 Department of Materials Engineering and Convergence Technology, Gyeongsang National University, Jinju 52828, Republic of Korea; 2 Composites Materials Application Research Center, Korea Institute of Science and Technology, 92 Chudong-ro, Bongdong-eup, Wanju-gun 55324, Jeonbuk, Republic of Korea; 3 School of Chemical Engineering, Pusan National University, Busan 46421, Republic of Korea

**Keywords:** polymer electrolyte membrane fuel cell, polymer electrolyte membrane, organic/inorganic composite membrane, composite materials, carbon nanotubes, graphene oxides, silica

## Abstract

To mitigate the dependence on fossil fuels and the associated global warming issues, numerous studies have focused on the development of eco-friendly energy conversion devices such as polymer electrolyte membrane fuel cells (PEMFCs) that directly convert chemical energy into electrical energy. As one of the key components in PEMFCs, polymer electrolyte membranes (PEMs) should have high proton conductivity and outstanding physicochemical stability during operation. Although the perfluorinated sulfonic acid (PFSA)-based PEMs and some of the hydrocarbon-based PEMs composed of rationally designed polymer structures are found to meet these criteria, there is an ongoing and pressing need to improve and fine-tune these further, to be useful in practical PEMFC operation. Incorporation of organic/inorganic fillers into the polymer matrix is one of the methods shown to be effective for controlling target PEM properties including thermal stability, mechanical properties, and physical stability, as well as proton conductivity. Functionalization of organic/inorganic fillers is critical to optimize the filler efficiency and dispersion, thus resulting in significant improvements to PEM properties. This review focused on the structural engineering of functionalized carbon and silica-based fillers and comparisons of the resulting PEM properties. Newly constructed composite membranes were compared to composite membrane containing non-functionalized fillers or pure polymer matrix membrane without fillers.

## 1. Introduction

**Figure 1:**
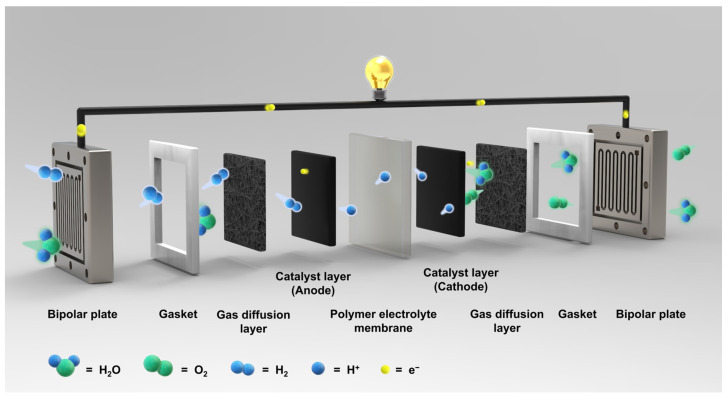
Schematic diagram of polymer electrolyte membrane fuel cells (PEMFCs).

**Figure 2:**
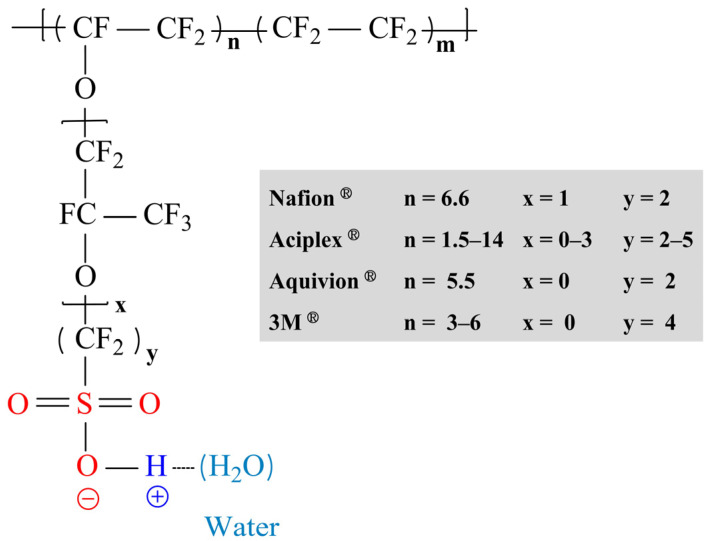
Types and structures of perfluorinated sulfonic acid polymers. Adapted with permission from [5]. Copyright 2017, Chemical Society.

The polymer electrolyte membrane fuel cell (PEMFC) is considered one of the most promising fuel cell systems due to its high efficiency and fast start-up [1]. During the operation of PEMFCs, hydrogen as a fuel, is oxidized to protons and electrons at the anode, and the electrons then move through an external circuit to generate electricity. At the same time, protons are transported through a polymer electrolyte membrane (PEM) to the cathode, and water is produced by a reduction reaction with oxygen fed at the cathode (Figure 1) [2]. Among the PEMFCs components, PEMs which serve as separators as well as an electrolyte that selectively transports protons from the anode to the cathode, have been considered a key component. To achieve outstanding PEMFC performance during long-term operation, PEMs need to have high proton conductivity and superior physicochemical stability. Currently, perfluorinated sulfonic acid (PFSA)-based PEMs, such as Nafion^®^ (DuPont), Gore-Select^®^ (Gore), Flemion^®^ (Asahi Glass), Aciplex^®^ (Asahi Chemical), Aquivion^®^ (Solvay), and Fumapem^®^ (Fumatech) have been applied in commercial PEMFC systems [3,4,5]. The reasons underlying the use of PFSA polymers (shown in Figure 2) are; (1) the hydrophobic perfluoro backbone with strong C-F bonding force, allows high physicochemical stability under harsh operating conditions; and (2) the flexible side chains containing highly acidic fluorosulfonic acid groups lead to high proton conductivity even under low humidity conditions by facilitating ionic cluster formation through a phase separated structure between the hydrophobic polymer backbone and hydrophilic side chain [6,7]. Despite these attributes, these PEMs have a number of shortcomings including limited operating temperatures due to their low glass transition temperatures, significant manufacturing costs, and severe gas cross-over [8,9].

A number of studies are underway to address these drawbacks, including some that are investigating the application of hydrocarbon-based PEMs to PEMFCs. Hydrocarbon-based PEMs are found to have high thermal stability and mechanical strength because the chemical structures of the polymer backbones are similar to well-known engineering plastics and are revealed to have low gas permeability given their high crystallinity [10,11,12]. Furthermore, the synthetic process is relatively simple compared to that of the PFSA polymers, and the polymer structure can be easily functionalized, allowing tunable structures while keeping the manufacturing costs low. However, sulfonated poly(arylene ether sulfone) (SPAES), sulfonated poly(ether ether ketone) (SPEEK), and sulfonated polyimide (SPI), which are well-known hydrocarbon polymers, are composed primarily of aromatic rings, and their respective main chains are not flexible enough to form ionic clusters by chain segmental motion (Table 1) [2]. In addition, since the proton conducting groups of those polymers (i.e., sulfonic acid groups) are directly attached to the stiff main chains, the formation of ionic clusters by the hydrophobic/hydrophilic phase separation is more difficult, and thus the proton conductivity of the hydrocarbon-based PEMs is relatively lower than that of PFSA-based PEMs. Accordingly, research has been conducted to improve proton conductivity by increasing the degree of sulfonation (DS) of the hydrocarbon polymers. However, water uptake of PEMs increases with the DS, which leads to degradation of physical stabilities (e.g., dimensional stability and mechanical strength) and to limit their application in PEMFCs due to the excessive swelling-deswelling behavior under actual operating conditions [13,14]. Another downside to hydrocarbon-based PEMs is low chemical stability due to the chemically unstable heteroatoms located in polymer backbones which are prone to attack by reactive oxygen species (ROS) produced during the fuel cell operation [15,16].

To resolve the aforementioned issues associated with both PFSA- and hydrocarbon-based PEMs, various studies have reported on the control of respective polymer architectures, the introduction of pore-filling or cross-linking concepts during the PEM fabrication, and the incorporation of organic/inorganic composite materials for the development of composite membranes [17,18,19,20,21,22]. Among these, the incorporation of composite materials is regarded as one of the simpler and more effective strategies for enhancing the original properties of respective pure PFSA-based or hydrocarbon-based PEMs. The incorporation of carbon nanomaterials, such as carbon nanotubes (CNTs), graphene, and graphene oxides (GOs) has also been reported as a means of creating alternative composites to improve the physicochemical and thermal stability of their corresponding polymer matrix membranes [23,24,25,26,27,28]. Furthermore, composite membranes using inorganic composites including silica (SiO_2_), titanium dioxide (TiO_2_), cerium dioxide (CeO_2_), zirconium dioxide (ZrO_2_), and montmorillonite (MMT) into the polymer matrix have been also reported to increase the water absorption and retention behavior, as well as physical stability of the pure polymer matrix membranes [29,30,31,32,33,34,35,36,37,38].

**Figure 3:**
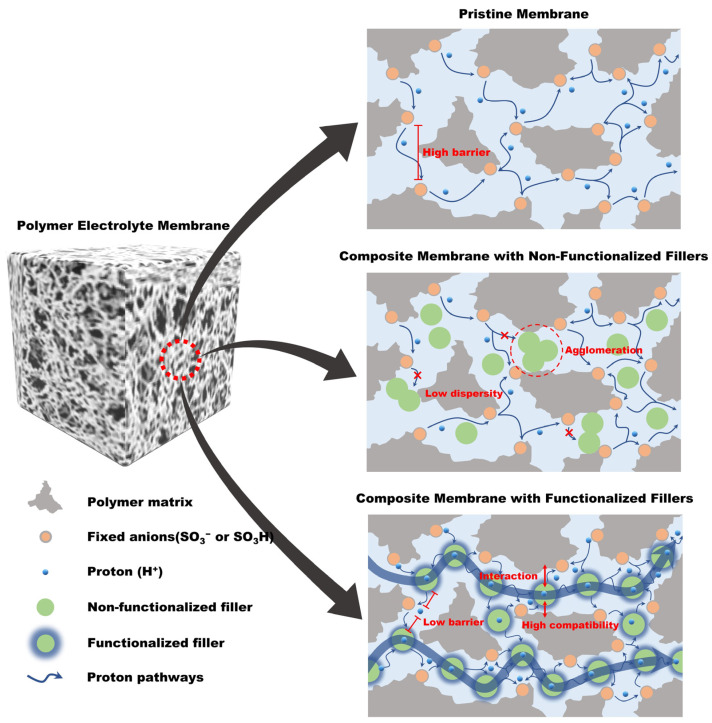
Schematic illustration of ion-conducting channel formation of a pristine membrane, a composite membrane with non-functionalized filler, and a composite membrane with functionalized filler, respectively.

Nevertheless, the dispersion characteristics of the composites are still problematic. When the chemically or physically non-functionalized composites are introduced into the polymer matrix, the agglomerated domains of composites are easily observed due to the low compatibility between the non-functionalized composites and the polymer matrix, resulting in impairment of PEM properties including mechanical strength, chemical stability, and proton conductivity [39]. To resolve these problems, studies have been conducted on the surface modifications to the composites via chemical treatment and/or grafting of functional groups or polymers. The aim being to increase the interfacial compatibility with the polymer matrix, resulting in enhanced PEM properties by synergistically increasing the interfacial interaction (Figure 3) [40,41,42,43,44,45]. Therefore, this review focused on recent trends in the development of high-performance composite membranes used in PEMFCs through incorporating functionalized fillers. The detailed modification procedures of functionalized CNT, GO, and SiO_2_ were also described. To the best of our knowledge, this is the first review to summarized the respective strategies for the development of PFSA- and hydrocarbon-based composite membranes by incorporating functionalized composites. 

## 2. Composite Membranes Used in PEMFCs

### 2.1. Composite Membranes with Functionalized Carbon Nanotubes

Carbon isotopes have been gaining significant attention in various fields given their abundant availability, easy processability, excellent stability, and environmental adaptability [46]. In particular, CNT, graphene, and GO are widely used as functional fillers to improve the performance of polymer composites [47]. CNTs have a structure, in which carbon atoms form a long cylindrical shape through the sp^2^ covalent bonding with graphite/graphene sheets rolled into tubes with nano scale diameters [48]. Depending on the number of sheets, CNTs can be classified as single-walled carbon nanotubes (SWCNTs), double-walled carbon nanotubes (DWCNTs), and multi-walled carbon nanotubes (MWCNTs) [49]. CNTs exhibit excellent thermal, mechanical, and electrical properties, and low weight density [50,51]. Given these characteristics, they have been extensively tested for use as fillers for polymer composites to improve performances in various applications [52,53,54,55,56,57,58,59,60]. However, since the aspect ratio of CNTs is very high, the Van der Waals forces between intermolecular CNTs is significant. Therefore, CNTs without physical or chemical treatment tend to aggregate with each other, resulting in low dispersion behavior and poor compatibility with polymer matrices when they are introduced in polymer composite systems [61,62,63,64]. To solve this problem, a number of organic functional groups or functional polymers have been introduced to the CNT surfaces to improve not only compatibility with the polymer matrix, but also PEM properties including proton conductivity, physical stability, and chemical stability [65,66,67,68,69,70,71,72,73,74,75].

#### 2.1.1. PFSA-Based Composite Membranes with Functionalized Carbon Nanotubes

**Figure 4:**
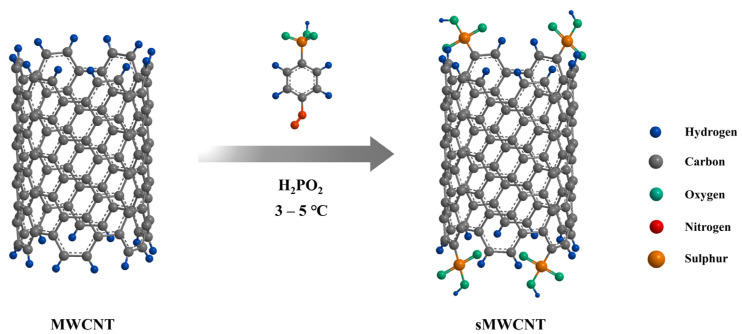
Schematic representation of sulfonation process by using sulfonic acid containing aryl radicals to introduce sulfonate groups on the surface of MWCNT. Adapted with permission from [76]. Copyright 2018, Elsevier.

To address the limitations of pure PFSA-based PEMs in PEMFC systems as described in the introduction, studies have reported improvements in performance and durability characteristics by preparing composite membranes with functionalized CNTs. Representatively, N. J Steffy et al. fabricated Nafion/sulfonated multi-walled carbon nanotube (sMWCNT) composite membranes using sMWCNT as a proton conducting filler prepared by grafting 4-benzendiazonium sulfonic acid onto the surfaces of MWCNT (Figure 4) [76]. The chemical structure and the content of sulfonic acid groups of sMWCNT were confirmed by X-ray powder diffraction (XRD), raman spectroscopy, energy dispersive X-ray spectroscope (EDS), field emission scanning electron micro-scope (FE-SEM) and transmission electron microscopy (TEM). The Nafion/sMWCNT composite membrane (23.0 mS cm^−1^) showed 11-fold higher proton conductivity value than recast Nafion (2.0 mS cm^−1^) under high-temperature and low relative humidity conditions of 80 °C and 20% RH (Table 2). This was due to the incorporation of additional sulfonic acid groups in sMWCNT increasing the water absorption behavior, while sMWCNT promotes the formation of ion conducting channels with the sulfonic acid groups in the Nafion matrix. When the content of sMWCNT was increased to greater than 0.5 wt.%, however, both the water uptake and proton conductivity values of the composite membranes were smaller than those of the recast Nafion, due to the agglomeration behavior of sMWCNT. These results indicated that the addition of an optimized content of sMWCNT containing the aryl sulfonic acid groups could enhance the PEM properties of Nafion-based composite membrane system by increasing the compatibility and thereby forming effective proton conducting channels. 

**Figure 5:**
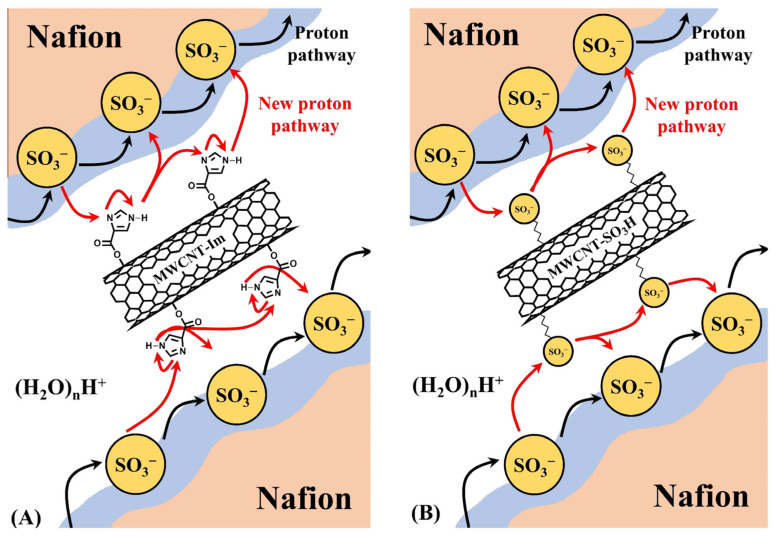
Schematic of the tortuous pathways of protons through (**A**) Nafion/MWCNT-Im and (**B**) Nafion/MWCNT-SO_3_H composite membranes. Adapted with permission from [77]. Copyright 2018, John Wiley and Sons.

Mahdi Tohidian et al. introduced imidazole and sulfonic acid groups on the surface of MWCNTs, respectively, to prepare imidazole-MWCNT (MWCNT-Im) and sulfonated-MWCNT (MWCNT-SO_3_H). Each of these composites was added to Nafion dispersions to develop Nafion/MWCNT-Im and Nafion/MWCNT-SO_3_H composite membranes (Figure 5) [77]. The water uptake and proton conductivity of the Nafion/MWCNT-SO_3_H composite membranes were higher than those of the recast Nafion due to the additional sulfonic acid groups. Although the basic imidazole groups possibly decrease the ion exchange capacity (IEC) of the membrane introduced in the composite membrane system, the Nafion/MWCNT-Im composite membrane revealed better proton conductivity than the recast Nafion due to enhanced proton transportation by the acid-base interaction between the positively charged imidazole groups on the surface of the MWCNT and the negatively charged sulfonic acid groups in Nafion forming proton hopping channels through the Grotthuss mechanism. Notably the Nafion/MWCNT-Im composite membrane showed the highest proton conductivity and physical stability (e.g., swelling ratio and mechanical strength) among the prepared samples. These results demonstrated that well-dispersed basic functional groups in an acidic polymer matrix using MWCNT can effectively increase the proton conductivity but also physical stability.

#### 2.1.2. Hydrocarbon-Based Composite Membranes with Functionalized Carbon Nanotubes

**Figure 6:**
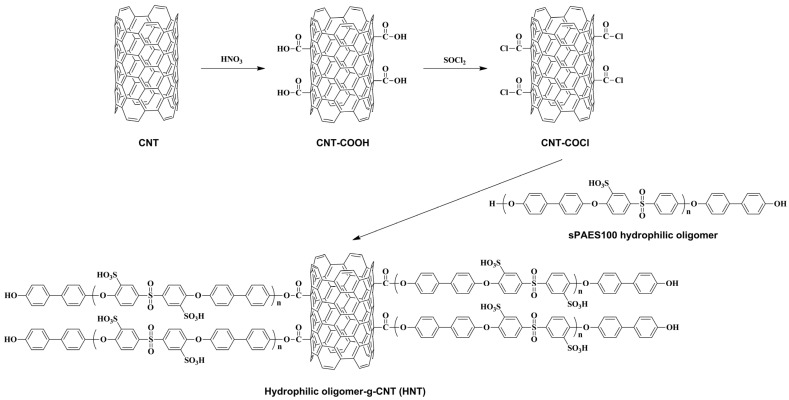
Synthesis of hydrophilic oligomer-g-CNT [78].

The major problems with hydrocarbon-based PEMs in PEMFC applications are the rapid drop of proton conductivity under low RH conditions (≤50% RH) and the deterioration of physicochemical properties (e.g., mechanical strength, swelling ratio, and oxidative stability) as the degree of sulfonation is increase. In order to improve these PEM properties, many studies have been conducted on the incorporation of functionalized carbon-based fillers into the hydrocarbon-based polymers. Representatively, In Hyouk Sung et al. reported the effect of a hydrophilic oligomer grafted CNT on sulfonated hydrocarbon-based composite membrane used in a PEMFC system [78]. A hydrophilic oligomer, sulfonated poly(arylene ether sulfone) with DS of 100 mol% (SPAES100), was synthesized by condensation polymerization using sulfonated dihalo monomer and biphenol. Then, SPAES100 was grafted on CNT by nucleophilic substitution to prepare hydrophilic oligomer-graft-CNT (HNT) (Figure 6). SPAES with DS of 50 mol% (SPAES50) was prepared as the hydrocarbon-based polymer matrix in the composite membrane system, and the HNT impregnated composite membrane (SPAES/HNT) was prepared by general solvent casting method via dispersing the optimal weight content of HNT in the SPAES50 solution. Since the SPAES100 oligomer in HNT has a similar structure with the polymer matrix (SPAES50), the compatibility issues inducing decrements in PEM properties were not found in SPAES/HNT membrane compared to the SPAES/CNT membrane. Furthermore, the low proton conductivity of general hydrocarbon-based PEMs at high temperature and low RH condition is improved considerably by the addition of HNT. The proton conductivity value of the SPAES/HNT membrane was about 38% larger than that of the pure SPAES50 membrane at 80 °C and 50% RH by forming additional ion conducting channels between HNT and SPAES50. The increased water uptake of the SPAES/HNT membrane also supported better formation of ionic clusters compared to the pure SPAES50 membrane. These results demonstrated that preparation of functional fillers having a similar structure to the polymer matrix can effectively increase the dispersity and thus enhance PEM properties.

**Figure 7:**
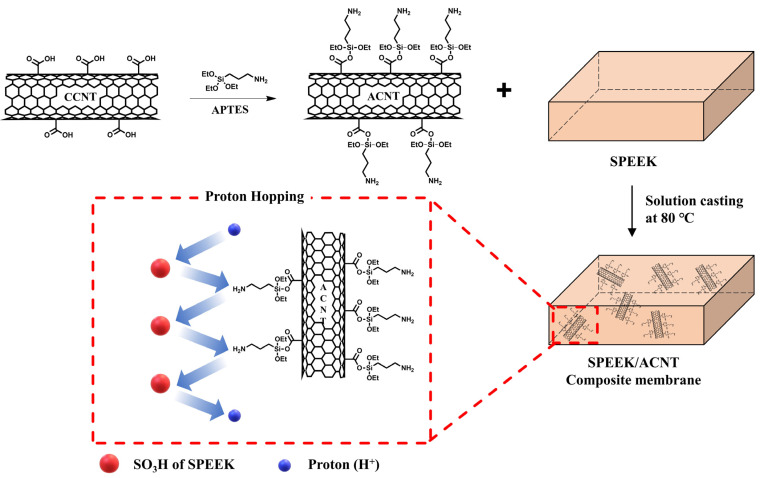
Preparation of SPEEK/ACNT composite membrane. Adapted with permission from [79]. Copyright 2020, Elsevier.

Ae Rhan Kim et al. prepared amine-functionalized CNT (ACNT) by grafting 3-aminopropyltriethoxysilane onto CNT with high carboxylic acid content (CCNT), through a condensation reaction. Then, two different types of SPEEK-based composite membranes were prepared by using ACNT and CNT as fillers to investigate the effect of ACNT on hydrocarbon-based composite membrane systems (Figure 7) [79]. As expected, CNT without any modification was found to aggregate due to the strong intermolecular Van der Waals forces [80,81]. On the other hand, ACNTs exhibited high dispersity when prepared in aprotic solvents due to the electrostatic repulsion caused by the amine (-NH_2_) groups [82]. The presence of electrostatic interactions between the amine groups of ACNT and the sulfonic acid groups of the SPEEK matrix, further improved the compatibility in the SPEEK/ACNT composite membrane compared to the SPEEK/CNT composite membrane. Therefore, the SPEEK/ACNT composite membrane showed much improved thermal and mechanical stability compared to the pure SPEEK membrane, although the SPEEK/CNT composite membrane exhibited worse properties. Furthermore, as described for MWCNT-Im in Section 2.1.1, similar proton conductivity behavior was observed when ACNT with basic amine groups introduced. The addition of ACNT improved the proton conductivity of the SPEEK-based PEM about 2-fold under high temperature and low RH conditions (80 °C and 20% RH) by creating and an additional proton hopping site that can reduce the activation energy for proton transport. These results indicated that the incorporation of CNT with basic functional groups was also an effective strategy for hydrocarbon-based composite membrane systems. The representative PEM properties including IEC, water uptake and proton conductivity of the composite membranes described in above are summarized in Table 3.

### 2.2. Composite Membranes with Functionalized Graphene Oxides

GO, which is a chemically functionalized graphene having sp^2^-hybridized structures and two-dimensional monolayer lattices can be applied in PEMFC systems due to its high rigidity and thermal stability as well as the radical scavenging effect produced by the presence of lattice defects such as reactive oxygen functional groups [83]. Furthermore, since GO has hydrophilic oxygen functional groups with a large surface area, a better proton conductivity of the GO-based composite membranes could be obtained when GO was well-dispersed [25,84,85,86]. Nevertheless, many studies have reported on the poor dispersion problem associated with pure GO when using organic solvent, resulting in agglomeration of GO into the polymer matrix even at low GO content (<1 wt.%) [87,88]. This phenomenon rather interferes with the formation of proton conductive channels and may degrade the physical properties of the composite membrane. To resolve this problem, research has been conducted to improve compatibility with the polymer matrix by attaching various functional groups including proton conducting acid groups on the GO surfaces, or by grafting polymers having similar structures to the polymer matrix to fabricate high-performance GO-based composite membranes [89,90,91,92,93,94,95].

#### 2.2.1. PFSA-Based Composite Membranes with Functionalized Graphene Oxides

**Figure 8:**
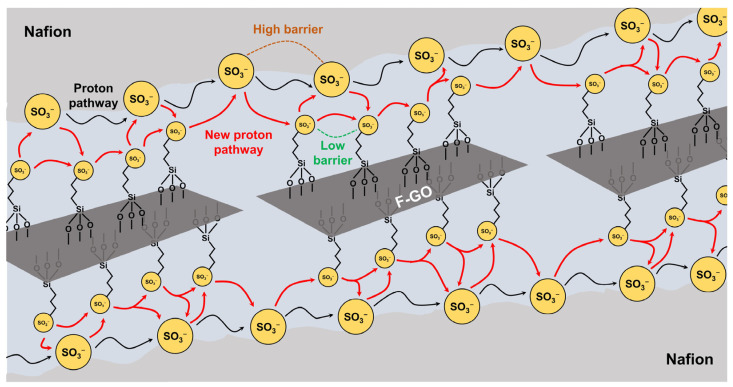
Schematic diagram of tortuous pathways of protons through Nafion/F-GO membrane [84].

Hadis Zarin et al. prepared functionalized GO (F-GO) via grafting 3-mercaptopropyl trimethoxysilane (MPTMS) on GO surfaces followed by sulfonation using 30 wt.% hydrogen peroxide solution. (Figure 8). Then, F-GO was impregnated into Nafion at different weight ratios to fabricate a series of Nafion/F-GO composite membranes [84]. The water uptake of the Nafion/F-GO composite membranes was 6.0% higher than that of recast Nafion due to the increase in the hydrophilic properties as the F-GO content increased. The proton conductivity of the Nafion/F-GO composite membranes was also larger than that of recast Nafion. In particular, due to the formation of additional proton conducting channels by the addition of F-GO, the proton conductivity of the composite membrane (8.0 mS cm^−1^) exhibits 4-fold higher than that of recast Nafion (2.0 mS cm^−1^) at 80 °C and 20% RH. Since F-GO with grafted sulfonic acid groups were well-dispersed in Nafion, the Nafion/F-GO membrane showed better overall PEM properties including physical stability, mechanical strength and proton conductivity, compared to the recast Nafion.

**Figure 9:**
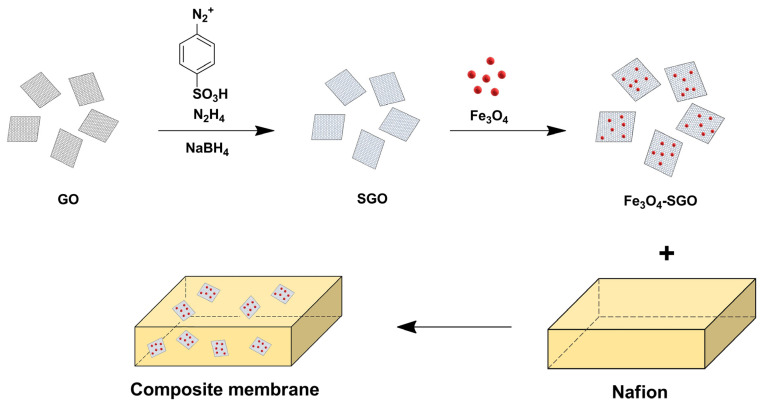
Preparation of Nafion/Fe_3_O_4_-SGO membrane. Adapted with permission from [96]. Copyright 2018, Royal Society of Chemistry.

Mohanraj Vinothkannan et al. synthesized sulfonated graphene oxide (S-GO) by grafting sulfanilic acid on the surface of GO, and then Fe_3_O_4_ containing a lot of hydrophilic hydroxyl (-OH) groups were dispersed in S-GO to develop Fe_3_O_4_-SGO. After that, a Nafion/Fe_3_O_4_-SGO composite membrane was prepared (Figure 9) [96]. The thermal stability and mechanical strength of the composite membrane were better than those of pure Nafion due to the strong interfacial interaction between the functional moieties of Fe_3_O_4_-SGO including -OH, epoxy (-O-), carboxylic acid (-CO_2_H), and sulfonic acid (-SO_3_H) groups and the hydrophilic linkages of Nafion as well as the introduction of thermo-mechanically stable inorganic Fe_3_O_4_ [97,98]. Furthermore, the composite membrane revealed better proton conductivity than the pure Nafion at high temperature over the entire range of RH conditions. In particular, the composite membrane revealed 4.6-fold higher proton conductivity than pure Nafion (2.5 mS cm^−1^) at 120 °C and 20% RH. Better performance arises because the sulfonic acid groups on S-GO effectively increased the bound water content at low RH conditions, thereby increasing the number of protons transported in the composite membranes through the Grotthuss mechanism, while the hydroxyl groups in Fe_3_O_4_ increase the free water content that can facilitate proton transport through the Vehicle mechanism [99,100,101]. The cell performance of membrane electrode assemblies (MEAs) with the composite membrane was about 1.9-fold larger than that with pure Nafion (0.11 W cm^−2^) at 120 °C and 25% RH due to the improved proton conductivity and decreased hydrogen cross-over as a consequence of the enhanced thermomechanical stability.

#### 2.2.2. Hydrocarbon-Based Composite Membranes with Functionalized Graphene Oxides

**Figure 10:**
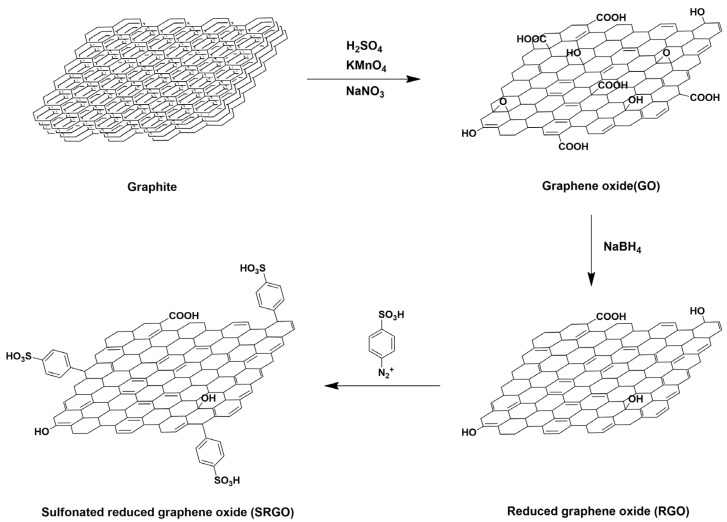
Synthesis of sulfonated reduced graphene oxide (SRGO). Adapted with permission from [102]. Copyright 2016, Elsevier.

**Figure 11:**
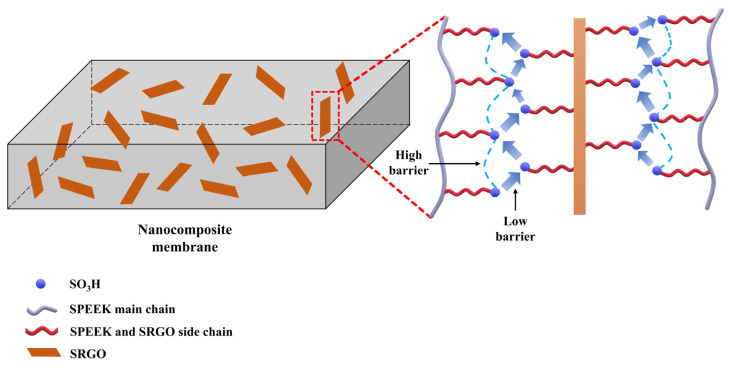
Formation of additional proton conducting channels between polymer matrix (SPEEK) and SRGO. Adapted with permission from [102]. Copyright 2016, Elsevier.

Xiang Qiu et al. prepared sulfonated reduced graphene oxide (SRGO) by grafting benzenesulfonic acid to reduced GO with diazonium salt through arylation (Figure 10), which was then added as a filler to sulfonated poly(ether ether ketone) (SPEEK) to develop the SPEEK/SRGO composite membrane [102]. The composite membrane exhibited three times higher proton conductivity than the pure SPEEK membrane at 80 °C and 50% RH condition. This occurs because, (1) the increased content of sulfonic acid groups from incorporation of SRGO allowed more absorption of water molecules to the composite membrane, and (2) well-dispersed SRGO with large sulfonic acid group content effectively reduces the proton transport barrier by forming additional proton conducting channels (Figure 11). In addition, the composite membrane exhibited 1.2-fold higher water uptake than the pure SPEEK membrane, but the swelling ratio was 4-fold smaller in the composite membrane due to the formation of hydrogen bonds between SRGO and SPEEK [103]. These results indicated that SRGO is one of the more effective fillers for resolving the problems of conventional hydrocarbon-based PEMs by increasing the proton conductivity at low RH conditions as well as increasing the physical stability of the composite membrane. 

**Figure 12:**
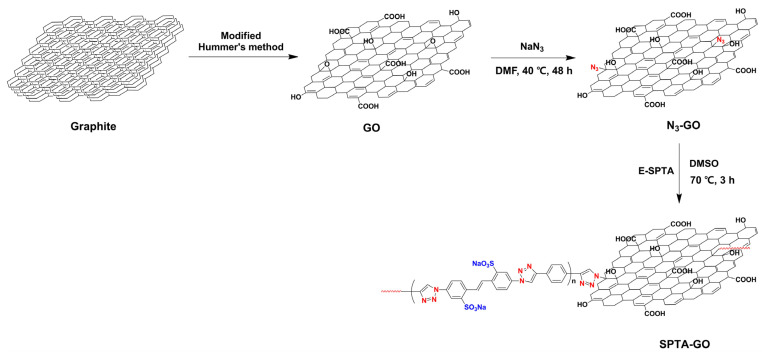
Preparation of sulfonated polytriazole graphene oxide (SPTA-GO). Adapted with permission from [104]. Copyright 2020, Elsevier.

Jusung Han et al. synthesized sulfonated polytriazole graphene oxide (SPTA-GO) through the azide-alkyne click reaction between GO with azide groups (N_3_-GO) and ethynyl-terminated sulfonated polytriazole (E-SPTA) (Figure 12). Then, a sulfonated poly(arylene ether sulfone) (SPAES)/SPTA-GO composite membrane was prepared by use of the well-known mixing process with SPTA-GO and SPAES [104]. The introduction of SPTA-GO was found to improve various PEM properties of the SPAES/SPTA-GO composite membrane. The oxidative stability of the composite membrane is better than that of the pure SPAES membrane due to the inherent radical scavenging effect of GO [105,106]. The mechanical strength of the composite membrane is also superior to that of the pure SPAES membrane due to the intermolecular ionic cross-linking between the basic triazole moieties in SPTA-GO and acidic sulfonic acid groups in SPAES. Notably, the elongation behavior of the composite membrane is also improved by the toughing effect from the acid-base interaction between the SPTA-GO and SPAES [107]. The proton conductivity of the composite membrane is larger than that of the SPAES membrane over the entire range of RH conditions due to the formation of additional proton conducting channels by the incorporation of basic triazole groups, which can function as proton donors and acceptors [108,109]. Since the physicochemical properties as well as the proton conductivity of the SPAES membrane was improved by the addition of SPTA-GO, outstanding MEA performance with the SPAES/SPTA-GO membrane (1.58 W cm^−2^) was obtained at operating condition (80 °C and 100% RH). The PEM properties of the above-described composite membranes with functionalized GOs are summarized in Table 4.

### 2.3. Composite Membranes with Functionalized Silica Composites

Numerous studies have been conducted on the incorporation of hygroscopic inorganic fillers such as SiO_2_, TiO_2_, CeO_2_, ZrO_2_, and MMT to the polymer matrix to improve water retention/management as well as proton conductivity of composite membranes used in PEMFCs operating at high temperature and low RH condition [112,113,114,115,116]. In particular, SiO_2_-based fillers for PEM applications have been studied intensively due to their hygroscopic nature, high thermal stability and relatively lower unit price compared to other inorganic particles [117,118,119,120,121]. Since the SiO_2_-based fillers are found to have high surface areas that possibly absorb and retain water molecules effectively, the great advantage of these fillers is preventing moisture evaporation to some extent even at high temperature and low RH conditions [122]. However, limited dispersion problems have been also reported when SiO_2_ without physical or chemical modifications were added to various polymer matrices used in PEMFCs [123]. In addition, when the particle size of non-functionalized SiO_2_ is larger than the size of ionic clusters formed by the hydrophilic/hydrophobic phase separation of the polymer matrix, the proton conducting channels were blocked by SiO_2_ resulting in the degradation of proton conductivity of the corresponding composite membranes [124]. To address these problems, many approaches have been investigated for functionalization of SiO_2_ [125,126,127]. 

#### 2.3.1. PFSA-Based Composite Membranes with Functionalized Silica

G. Ganna Kumar et al. synthesized silica sulfonic acid with particle sizes of 3, 90, and 1000 nm, respectively, through the general sulfonation reaction using chlorosulfonic acid. Then, the fillers were introduced into Nafion dispersions to develop Nafion/silica sulfonic acid membranes (NSSH-X, with X indicating the size of the silica sulfonic acid, X = 3, 90, 1000 nm) [128]. Nafion membranes containing non-functionalized silica with the same particle sizes of silica sulfonic acid (NS-X) were also prepared for comparison. The rank order for water uptake of the membranes was found to be NSSH-X > NS-X > pure Nafion due to the hygroscopic and porous characteristic of the silica-based fillers. Comparing each of the composite membranes, the NSSH-X membranes showed 1.2-fold larger water uptake values than the NS-X membranes when they contained the same size particles due to the enhanced IECs through the addition of the acid-containing silica. Therefore, the conductivity of the NSSH-X membranes was found to be better than that of the NS-X membrane over the entire range of RH conditions, due to the increased IEC and better formation of ion-conducting channels by sulfonic acid groups in silica when the composite membranes contained the same size particles. However, the proton conductivities of each type were highly affected by the particle size. As the size increased, the proton conductivity of the corresponding composite membranes decreased even though the silica sulfonic acid was incorporated. For example, the proton conductivity of NSSH-90 was lower than that of pure Nafion because particle sizes of 90 nm may block the 4–5 nm ionic clusters formed by the hydrophilic/hydrophobic separation of Nafion. For the NSSH-1000 membrane, the deterioration of proton conductivity was even larger than that seen with NSSH-90. However, under high temperature and low RH conditions of 80 °C and 30% RH, the proton conductivity of the NSSH-3 membrane (49.1 mS cm^−1^) was 12-fold better than that of pure Nafion (4.2 mS cm^−1^) due to the hygroscopicity of the silica-based filler. This study systematically investigated the importance of the size of inorganic fillers for application in PEMs (Table 5).

In a similar study, Kwangjin Oh et al. prepared sulfonated silica (SSA) as a functional filler for PFSA-based PEM. SSA was synthesized via hydrolysis of orthosilicate (TEOS) followed by sulfonation using chlorosulfonic acid. The chemical structure and the content of sulfonic acid groups of SSA were confirmed by fourier transform infrared (FT-IR), X-ray photoelectron spectroscopy (XPS), and TEM. The product was then dispersed in PFSA dispersion, and a general solution casting method was conducted to fabricate PFSA/SSA composite membranes [122]. The incorporation of SSA into PFSA increased the mechanical strength while decreasing the elongation behavior of the composite membrane because the SSA inorganic filler inhibited the segmental motion of polymer chains. AS well, the issue of low thermal stability of PFSA-based PEMs could be addressed by adding SSA, due to the high thermal stability of SSA as well as the barrier effect of SSA on heat transfer in the composite membrane system. The proton conductivity was also improved by the addition of SSA, due to the formation of additional proton conducting channels with PFSA (Table 6). These results indicated that SSA is an effective inorganic filler for PFSA-based PEMs through its ability to increase both the physicochemical stability and the proton conductivity, simultaneously.

#### 2.3.2. Hydrocarbon-Based Composite Membrane with Functionalized Silica

**Figure 13:**
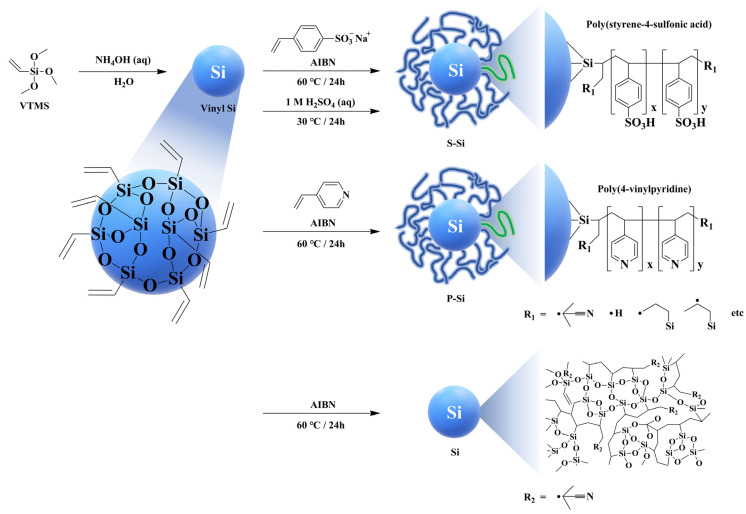
Preparation of silica-based composite materials. Adapted with permission from [126]. Copyright 2015, Elsevier.

Taeyun Ko et al. synthesized vinyl silica (vinyl Si) from vinyltrimethoxysilane through a hydrolysis reaction and then radical polymerization was conducted using each of 4-styrensulfonic acid sodium salt hydrate (SSANa) and 4-vinylpyridine (4VP) to prepare two different types of core–shell silica particles with poly(4-styrenesulfonic acid) (PSSA) and poly(4-vinylpyridine) (P4VP) in the shell layer, respectively. The acidic PSSA and basic P4VP grafted core–shell silica particles were named S-Si and P-Si, respectively (Figure 13) [126]. Following this, the SPAES based composite membranes with 5 wt.% of S-Si and P-Si were fabricated to investigate the effect of these functionalized fillers on PEM properties. As observed in most organic/inorganic composite membranes, the mechanical strength of the SPAES/S-Si and SPAES/P-Si composite membranes were greater than that of the pure SPAES membrane. Of note, the mechanical strength of the P-Si filler was demonstratively better than that of the S-Si filler due to the presence of ionic cross-linking between the basic P4VP in the shell layer of P-Si and the acidic sulfonic acid groups of the SPAES matrix [129]. Although the water uptake of the SPAES/S-Si and SPAES/P-Si composite membranes were lower than that of pure SPAES membrane, the proton conductivity of both composite membranes was larger than that of the SPAES membrane for two different reasons. For the SPAES/S-Si membrane, the enhanced proton conductivity could be ascribed to the increased sulfonic acid content, resulting in additional proton conducting channels with the sulfonic acid groups in the SPAES matrix. The increased IEC value calculated by acid-base titration method and change of hydrophilic domain size measured by small-angle x-ray scattering (SAXS), supported the enhanced proton conductivity of the SPAES/S-Si composite membrane. In contrast, the SPAES/P-Si membrane showed 2-fold better proton conductivity than the SPAES membrane (1.1 mS cm^−1^) at 80 °C and 40% RH due to the formation of additional proton hopping channels by the acid-based interactions between the pyridine groups in P-Si and the sulfonic acid groups in the SPAES matrix, resulting in the formation of protonation-deprotonation loops [109,130,131]. The calculation of activation energy of proton transport by Arrhenius plots, and comparison of SAXS data of SPAES/P-Si and pure SPAES membranes, also supported the enhanced proton conductivity of the SPAES/P-Si membrane.

**Figure 14:**
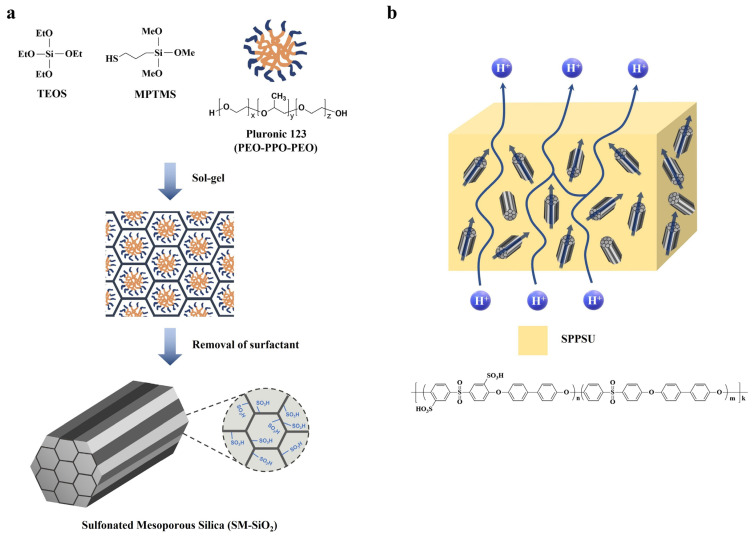
Schematic representations of (**a**) SM-SiO_2_ and (**b**) SPPSU composite membrane, wherein the conceptual proton transport pathway of the SPPSU composite membrane is also illustrated. Adapted with permission from [132]. Copyright 2012, Elsevier.

Jihye Won et al. prepared rod-shaped mesoporous silica (SBA-15) by using tetraethyl orthosilicate (TEOS) as a mechanical framework precursor and poly(ethylene glycol)-block-poly(propylene glycol)-block-poly(ethylene glycol) (Pluronic^®^ p-123) to form rod-shaped micelles as a mesopore-forming template for developing mesoporous structures [132]. Subsequently, they introduced a sulfonic acid moiety using mercaptopropyl trimethoxysilane (MPTMS) and H_2_O_2_ via condensation method followed by oxidation. Finally, the Pluronic^®^ p-123 template was removed from the synthesized material by washing with ethanol. The structure and acid content of rod-shaped mesoporous silica with sulfonic acid groups (named SM-SiO_2_) was confirmed by FT-IR, TEM, FE-SEM, XRD, and EDS. SM-SiO_2_ was incorporated into the sulfonated poly(phenylsulfone) (SPPSU) matrix to prepare SPPSU/SM-SiO_2_ composite membranes (Figure 14) with a weight ratio of SPPSU to SM-SiO_2_ of 95: 0.5. Although the water uptake of the SPPSU/SM-SiO_2_ composite membrane was larger than the pure SPPSU membrane, the proton conductivity value at 100% RH of the composite membrane was lower than that of the pure membrane due to the barrier effect of inorganic particles which interrupted the excessive swelling under fully hydrated condition [133]. The authors explained that the pure SPPSU membrane could be completely hydrated and contain excessive water at 100% RH conditions, therefore the proton-conducting hydrophilic domains could easily be connected, allowing protons to move quickly, while in the case of the SM-SiO_2_ composite membrane, the inorganic SM-SiO_2_ particles could block the connecting of hydrophilic domains by excess water, resulting in interruptions to proton conduction via a winding proton pathway. However, SM-SiO_2_ had positive effects on proton conductivity when observed under low RH conditions of 50% RH). The proton conductivity value of the SPPSU/SM-SiO_2_ composite membrane was 6.0 mS cm^−1^, Which is 1.6-fold larger than the proton conductivity value of the pure SPPSU membrane at 50% RH. This occurs because, (1) the mesoporous structure in addition to the sulfonic acid groups of the SM-SiO_2_ particles effectively attracted water molecules and then maintained the absorbed water molecules for an extended period; (2) chemically bound water molecules presented in the cylindrical hexagonal-mesopores of SM-SiO_2_ could effectively form proton-hopping channels under low RH conditions. This result indicates that rational design of inorganic fillers through control of physical structures, can effectively complement the disadvantages of hydrocarbon-based PEMs, including those of poor proton conductivity at low RH and excessive swelling when fully hydrated. The PEM properties of the above-described composite membranes with functionalized SiO_2_ are summarized in Table 7. Based on the literature studies, the optimized composite materials including types and content for PEMs were found to be highly dependent on the operating conditions of PEMFCs. For example, the functionalized SiO_2_-based composites effectively increased the proton conductivity of the corresponding composite membranes, especially at high temperature and low RH (≤40% RH) conditions due to the inherent water absorption and retention behavior of SiO_2_. Meanwhile, the functionalized GO-based composites led to the highest proton conductivity of the PEMs due to the highest compatibility with the polymer matrix preventing the excessive swelling under high RH conditions. To enable the PEMFC operation under a wide range of conditions that are off-limits for existing PFSA- or hydrocarbon-based PEMFCs, the novel organic/inorganic hybrid composite PEMs should be developed. One of the effective strategies for developing novel composites is to have complex structures of functionalized GO-SiO_2_ showing each advantage of organic and inorganic. In addition, the development of multifunctional fillers providing additional proton conducting channels as well as forming the covalently bonded framework with polymer matrices should be considered. The development of functionalized covalent organic frameworks having much larger surface areas and lots of functional groups than the commercialized fillers also could be a candidate to develop composite PEMs showing operational flexibility.

## 3. Conclusions

Incorporation of functionalized organic/inorganic materials into PFSA and hydrocarbon-based polymer matrices is a promising way of increasing the proton conductivity and physicochemical stability of PEMs used in PEMFCs. Functionalization of composite materials using various strategies should be prioritized when considering the improvement of target PEM properties as well as the enhancement of filler dispersity into the polymer matrix. Therefore, this review focused on both the design and the synthetic strategies underlying functionalized organic/inorganic fillers such as CNTs, GOs, and SiO_2_ and the development of their corresponding composite membranes that demonstrate improved PEM properties. For carbon-based fillers, most of the functionalization can be performed using the oxidated 1D or 2D carbon materials having unsaturated defects such as CNTs and GOs. The modification of oxygen functional groups in the CNTs and GOs through various chemical reactions has been mainly used for functionalizing and grafting the functionalized polymer onto the fillers. Since the optimized content of functionalized carbon-based fillers to PFSA and hydrocarbon-based polymer matrices is different than for non-functionalized composites due to the enhanced filler dispersity, highly improved PEM properties including thermal stability, mechanical strength, dimensional stability, and proton conductivity of the composite membranes are observed. In particular, the functionalized fillers effectively improve the proton conductivity of PEMs by forming additional proton conducting channels with the ionic domains of the polymer matrix. Therefore, the MEA performances employing these composite membranes are better than those employing the composite membranes containing non-functionalized fillers or pure polymer matrix membranes under various operating conditions. Considering the inorganic-based fillers, functionalized SiO_2_-based fillers have been widely used as effective composite materials for PEMs due to the inexpensive cost of the raw materials and simple modification processes. The hydrolysis of the silica precursor is mainly used for introducing functional groups on the SiO_2_ surfaces, while the various chemical reactions and pore-forming agents were used to increase the surface area of the fillers for increasing the degree of functionality. Acidic and basic functional groups were commonly introduced to the surface of the SiO_2_ based fillers to increase the water retention properties and proton conductivity of the composite membranes without deterioration of physical stability. The grafting of functionalized polymer containing both acidic and basic groups was also studied and found to increase the proton conductivity and physicochemical stability simultaneously, by forming protonation-deprotonation loops with the polymer matrix through enhanced intermolecular interactions. Based on this review of existing literature, we believe that functionalization of organic/inorganic fillers beginning with the synthetic process is an effective and facile way to tune the PEM properties of both PFSA and hydrocarbon-based PEMs for practical and wide application of PEMFCs.

## Figures and Tables

**Table 1:** Representative hydrocarbon-based polymer used in polymer electrolyte membrane fuel cells. Adapted with permission from [2]. Copyright 2020, KoreaScience.

Polymer	Structure
SPSf ^a^	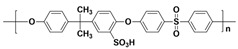
SPEEK ^b^	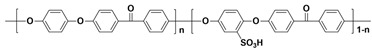
SPAES ^c^	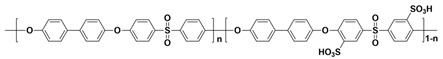
SPI ^d^	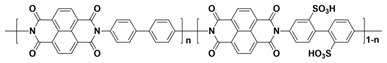

^a^ Sulfonated poly sulfone. ^b^ Sulfonated poly(ether ether sulfone). ^c^ Sulfonated poly(arylene ether sulfone). ^d^ Sulfonated polyimide.

**Table 2:** Water uptake and proton conductivity of membranes. Adapted with permission from [76]. Copyright 2018, Elsevier.

Sample	Water Uptake ^a^ (%)	Proton Conductivity ^b^ (mS cm^−1^)	
		20% RH	80% RH
Nafion/sMWCNT (0.125 wt.%)	17.2	8.0	180
Nafion/sMWCNT (0.25 wt.%)	29.3	23.0	198
Nafion/sMWCNT (0.5 wt.%)	16.4	6.0	165
Recast Nafion	15.8	2.0	160

^a^ Measured at room temperature. ^b^ Proton conductivity at 60 °C.

**Table 3:** Functionalization method of CNT via different reagents, and representative PEM properties of corresponding composite membranes.

Composite Membrane (Matrix/Filler)	Filler Content (wt. %)	Functionalization of CNTs		Water Uptake ^a^ (%)	IEC ^b^ (meq g^−1^)	Proton Conductivity ^c^ (mS cm^−1^)		Ref.
		Reagent	Method			20% RH	80% RH	
Nafion/CNT	5.0	-	-	20.7	0.8	1.0	40.0	[53]
Nafion/sMWCNT ^d^	0.25	4-benzendiazonium sulfonic acid	Grafting	29.3	-	2.3 (60 °C)	198 (60 °C)	[76]
Nafion/MWCNT-SO_3_H ^e^	0.5	1,3-propanesultone		34.5	0.9	-	150	[77]
Nafion/MWCNT-Im ^f^	0.5	4-imidazolecarboxylic acid	Dehydration reaction	28.0	0.9	-	210	[77]
SPEEK/CNT	1.5	-		-	-	2.8	47.0	[79]
SPAES/HNT ^g^	1.0	SPAES 100 hydrophilic oligomer	Nucleophilic substitution	58.0	-	0.7	237 (100% RH)	[78]
SPEEK/ACNT ^h^	1.5	3-aminopropyl triethoxysilane	Condensation reaction	17.5	1.2	7.3	115	[79]

^a^ Measured at room temperature. ^b^ Determined by titration at 25 °C. ^c^ Proton conductivity at 80 °C. ^d,e^ Sulfonic acid group grafted multi-walled carbon nanotube. ^f^ Imidazole group grafted multi-walled carbon nanotube. ^g^ Sulfonated poly(arylene ether sulfone) hydrophilic oligomer grafted carbon nanotube. ^h^ Amine group grafted carbon nanotube.

**Table 4:** Functionalization method of GO via different reagents, and representative PEM properties of corresponding composite membranes.

Composite Membrane (Matrix/Filler)	Filler Content (wt. %)	Functionalization of GOs		Water Uptake ^a^ (%)	IEC ^b^ (meq g^−1^)	Proton Conductivity ^c^ (mS cm^−1^)		Ref.
		Agent	Method			20% RH	80% RH	
Nafion/GO	5.0	-	-	57.5	0.8	2.0	79.0	[110]
Nafion/F-GO	10	3-mercaptopropyl trimethoxysilane	Oxidation	28.8	1.0	8.0	110	[84]
Nafion/Fe_3_O_4_-SGO	3.0	Sulfanilic acid, Fe_3_O_4_	Arylation, Dispersion	35.6 (60 °C)	1.4	7.0	125 (100% RH)	[96]
SPAES/GO	1.0	-	-	27.8	1.6	1.6 (50% RH)	114 (90% RH)	[111]
SPEEK/SRGO	1.0	4-aminobenzensulfonic acid	Arylation	31.1 (80 °C)	1.7	8.6 (50% RH)	147 (95% RH)	[102]
SPAES/SPTA-GO	1.0	Ethynyl-terminated sulfonated polytriazole	Azide-alkyne click reaction	57.0	2.5	20 (40% RH)	250	[104]

^a^ Measured at room temperature. ^b^ Determined by titration at 25 °C. ^c^ Proton conductivity at 80 °C.

**Table 5:** Water uptake, ion exchange capacity and proton conductivity of membranes. Adapted with permission from [128]. Copyright 2009, Elsevier.

Sample (SiO_2_ Particle Size)	Nomenclature	Water Uptake (%)	IEC (meq g^−1^)	Proton Conductivity ^a^ (mS cm^−1^)	
				30% RH	80% RH
Nafion 117	N	23.0	0.9	4.2	108
Nafion/SiO_2_ (3 nm)	NS-3	28.0	0.9	-	-
Nafion/SiO_2_ (90 nm)	NS-90	25.0	0.9	-	-
Nafion/SiO_2_ (1000 nm)	NS-1000	21.0	0.7	-	-
Nafion/SiO_2_-O-SO_3_H (3 nm)	NSSH-3	32.2	1.5	49.1	193
Nafion/SiO_2_ SiO_2_-O-SO_3_H (90 nm)	NSSH-90	30.0	1.3	30.9	180
Nafion/SiO_2_ SiO_2_-O-SO_3_H (1000 nm)	NSSH-1000	23.3	0.9	0.4	54.4

^a^ Proton conductivity at 80 °C.

**Table 6:** Ion exchange capacity, water uptake and proton conductivity of Nafion-SSA composite membranes. Adapted with permission from [122]. Copyright 2019, Elsevier.

Sample	IEC (meq g^−1^)	Water Uptake (%)	Proton Conductivity ^a^ (mS cm^−1^)	
			20% RH	80% RH
Nafion/SSA (0.5 wt.%)	1.2	22.0	5.9	151
Nafion/SSA (1.0 wt.%)	1.3	24.1	8.8	230
Nafion/SSA (1.5 wt.%)	1.1	20.0	5.5	143
Recast Nafion	1.0	17.3	5.0	111

^a^ Proton conductivity at 80 °C.

**Table 7:** Functionalization method of SiO_2_ via different reagents, and representative PEM properties of corresponding composite membranes.

Sample (Matrix/Filler)	Filler Content (wt. %)	Functionalization of SiO_2_		Water Uptake ^a^ (%)	IEC ^b^ (meq g^−1^)	Proton Conductivity ^c^ (mS cm^−1^)		Ref.
		Agent	Method			20% RH	80% RH	
Nafion/SiO_2_	2.0	-	-	35.6	-	7.5	80.0	[134]
Nafion/Silica sulfonic acid	-	Chlorosulfonic acid	Sulfonation	32.2	1.5	40.0	175	[128]
Nafion/SSA ^d^	1.0	TEOS, Chlorosulfonic acid	In situ sol-gel methods	24.1	1.3	8.8	230 (100% RH)	[122]
SPAES/SiO_2_	5.0	-	-	36.7	1.7	1.0 (40% RH)	102 (90% RH)	[126]
SPAES/S-Si ^e^	5.0	4-styrensulfonic acid sodium salt hydrate	Radical polymerization	40.9	1.7	1.3 (40% RH)	140 (90% RH)	[126]
SPAES/P-Si ^f^	5.0	4-vinylpyridine	Radical polymerization	34.3	1.6	2.3 (40% RH)	146 (90% RH)	[126]
SPPSU/SM-SiO_2_^g^	-	3-mercaptoproyl trimethoxysilane	Condensation, oxidation	17.0	2.0	6.0 (50% RH)	183 (100% RH)	[132]

^a^ Measured at room temperature. ^b^ Determined by titration at 25 °C. ^c^ Proton conductivity at 80 °C. ^d^ Silica sulfonic acid. ^e^ Poly(4-styrenesulfonic acid) grafted core-shell silica particles. ^f^ Poly(4-vinylpyridine) grafted core-shell silica particles. ^g^ Sulfonated SBA-15 mesoporous silica

## Data Availability

Not applicable.
